# Draft genomes of six methicillin-resistant *Staphylococcus aureus* in a Philippine tertiary hospital with insights on putative antimicrobial and phage resistance mechanisms

**DOI:** 10.1128/mra.00097-25

**Published:** 2025-02-25

**Authors:** Mark B. Carascal, Jo-Eliz M. Gonzales, Ma. Mona Joy T. Tañedo, Donna May D. Papa, Raul V. Destura, Karl Evans R. Henson

**Affiliations:** 1 Clinical and Translational Research Institute, The Medical City, Pasig City, Philippines; 2 Bacteriophage Ecology, Aquaculture, Therapy, and Systematics Laboratory, Research Center for Natural and Applied Sciences, University of Santo Tomas, Manila, Philippines; Queens College Department of Biology, Queens, New York, USA

**Keywords:** *Staphylococcus aureus*, methicillin resistance, bacteriophage resistance, draft genome

## Abstract

The genomes of six methicillin-resistant *Staphylococcus aureus* (MRSA) revealed CC5(ST5)-SCC*mec*-IVc-*spa*-t105-PVL^+^ (*n* = 3), CC5(ST6)-SCC*mec*-IVc-*spa*-t10002 (*n* = 1), CC5(ST5)-SCC*mec*-IVa-*spa*-t002 (*n* = 1), and CC8(ST8)-SCC*mec*-IVa-*spa*-t008-PVL^+^ (*n* = 1) genotypes. All possessed multiple antibiotic resistance genes (putative *mecA* and *blaZ,* and *dfrG* for cotrimoxazole-resistant strains), intact staphylococcal prophages, and putative antiphage systems. The results could aid in MRSA infection management and control.

## ANNOUNCEMENT

Methicillin-resistant *Staphylococcus aureus* (MRSA) remains a significant clinical threat in the Philippines, with recent annual reported occurrence of >40% and high rates of resistance to co-trimoxazole (1). Among the promising options to combat this pathogen are lytic bacteriophages (2, 3). However, phage resistance can also arise in highly virulent bacterial hosts (4). Thus, we analyzed the draft genomes of six clinical MRSA isolates from a Philippine tertiary hospital for the presence of putative antibiotic and phage resistance genes. The study protocol has been approved by The Medical City Institutional Review Board (GCS-Med-2023-028).

All MRSA were directly isolated from patient clinical samples (wound exudates [*n* = 3], respiratory samples [*n* = 2], blood [*n* = 1]) using blood agar (Thermo Scientific, USA) and purified in tryptic soy agar (TSA) (Merck, Germany). All cultures were incubated for 18–24 hours at 35℃–37°C under normal atmospheric conditions. All isolates were identified using Vitek MS Matrix-Assisted Laser Desorption-Ionization Time-of-Flight (bioMerieux, France) and tested for antibiotic resistance using Vitek 2 Compact with AST-GP67 card (bioMerieux, France). Testing was conducted at The Medical City hospital following the Clinical and Laboratory Standards Institute guidelines (5). The DNA from a single colony of overnight cultures of MRSA (in TSA) was extracted using the DNeasy Blood and Tissue kit with lysozyme (QIAGEN, Germany). Libraries were prepared using the TruSeq Nano DNA Library Preparation kit (Illumina, USA), and the genome was sequenced using NovaSeq 6000 (150bp, paired-end; Illumina, USA). Raw sequences were trimmed using BBMap v.39.01 (Q ≥28) (6), subsampled using Seqtk v.1.3-r106 (https://github.com/lh3/seqtk) to 5M reads (≈500× coverage), and *de novo* assembled using Unicycler v.0.5.1 (7). The quality and completeness of the assembled contigs were checked using QUAST v.5.2.0 (8) and CheckM v.1.2.3, (9) and the average coverage computed using BBMap v.39.01 (6). The assemblies were initially annotated using the Prokaryotic Genome Annotation Pipeline (10). Extended annotation and analysis were done using the *baargin* pipeline (11). SCC*mec*, *spa*, and multilocus sequence types were determined using SCC*mec*Finder v.1.2 (https://cge.food.dtu.dk/services/SCCmecFinder/), *spa*Typer v.0.2.1 (https://cge.food.dtu.dk/services/spaTyper/), and PubMLST (https://pubmlst.org/organisms/staphylococcus-aureus), respectively. Antibiotic resistance genes were predicted using AMRFinderPlus (12) in *baargin*, while prophages and the presence of known antiphage systems were inferred using PHASTEST (https://phastest.ca/) and DefenseFinder v.1.3.0 (13), respectively. Default parameters were used for the programs.

Table 1 shows that all MRSA isolates are resistant to at least two beta-lactam antibiotics. Three strains are also cotrimoxazole-resistant (TMCC-SA5, -SA6, -SA8), while one is quinolone-resistant (TMCC-SA1). The assembled genomes are between 2.73 and 2.82Mbp (97.35%–99.11% complete), with 32.63%–32.71% GC and at least 482× coverage. Contigs are between 27 and 40 with N_50_ >145 kbp, and predicted coding sequences between 2,514 and 2,655. Genotypes include CC5(ST5)-SCC*mec*-IVc-*spa*-t105-PVL^+^ (*n* = 3), CC5(ST6)-SCC*mec*-IVc-*spa*-t10002 (*n* = 1), CC5(ST5)-SCC*mec*-IVa-*spa*-t002 (*n* = 1), and CC8(ST8)-SCC*mec*-IVa-*spa*-t008-PVL^+^ (*n* = 1), all with putative *mecA* and *blaZ* (beta-lactam resistance). In comparison, the pioneer genome analysis of Philippine MRSA reported the dominance of CC30-SCC*mec*-IV-*spa*-t019-PVL^+^ (14). All cotrimoxazole-resistant strains (*n* = 3) possess putative *dfrG*. At least one intact staphylococcal prophage and four putative anti-phage systems (with Abi2 as the most common) were predicted for all isolates. The information gathered from this study could guide the local management of MRSA infections, whether using antibiotics or phage therapy.

**TABLE 1:** Phenotypes, genome assembly, and annotation characteristics of six methicillin-resistant *Staphylococcus aureus* clinical strains from a Philippine tertiary hospital^*a*^

Characteristics/ genome statistics	Methicillin-resistant *S. aureus* strains					
	TMCC-SA1	TMCC-SA3	TMCC-SA5	TMCC-SA6	TMCC-SA8	TMCC-SA18
Clinical isolation source	Pus	Nasal cavity	Blood	Abscess	Sputum	Wound
Antibiotic resistance profile (MIC^*b*^ values in mg/L)	Resistant to ciprofloxacin (≥4), levofloxacin (≥4), and oxacillin (≥4)	Resistant to benzylpenicillin (≥0.5) and oxacillin (≥4)	Resistant to benzylpenicillin (≥0.5), oxacillin (≥4), and cotrimoxazole (≥320)	Resistant to benzylpenicillin (≥0.5), oxacillin (≥4), and cotrimoxazole (≥320)	Resistant to benzylpenicillin (≥0.5), oxacillin (≥4), and cotrimoxazole (≥320)	Resistant to benzylpenicillin (≥0.5) and oxacillin (≥4)
Raw reads	16,769,261	21,507,160	17,900,325	17,208,057	19,513,731	19,688,234
Assembly length (bp)	2,821,009	2,760,446	2,791,932	2,820,064	2,820,985	2,733,769
GC content (%)	32.65	32.67	32.71	32.67	32.68	32.63
Longest contig (bp)	471,356	586,132	438,318	579,621	579,676	468,743
Number of contigs	40	34	31	37	34	29
N_50_ (bp)	201,442	145,206	152,015	173,209	151,856	290,936
Average genome coverage (×)	482.1	505.7	500.2	489.7	497.0	511.0
Completeness (%)	97.35	99.00	99.11	99.11	98.92	99.11
Contamination (%)	0.83	0.39	0.24	1.12	0.54	0.24
Coding sequences	2,655	2,540	2,608	2,647	2,653	2,514
rRNA	1	2	1	1	1	1
Plasmids	4	2	0	5	4	3
Multilocus sequence type	CC8 (ST8)	CC5 (ST6)	CC5 (ST5)	CC5 (ST5)	CC5 (ST5)	CC5 (ST5)
SCC*mec* type	IVa (2B)	IVc (2B)	IVc (2B)	IVc (2B)	IVc (2B)	IVa (2B)
*spa* type	t008	t10002	t105	t105	t105	t002
Antibiotic resistance genes	Beta-lactams: *mecA, blaZ, blaR1, blaI* Aminoglycosides: *aph(3′)-IIIa, kdpD* Quinolones: *gyrA, parC, norA, norC, sdrM, arlR, arlS* Tetracyclines: *mepA, mepR, tet(38*) Fosfomycin: *murA, fosB* Streptothricin: *sat4* Efflux/ Others: *mgrA, LmrS, sepA*	Beta-lactams: *mecA, blaZ, blaR1, blaI* Aminoglycosides: *kdpD* Quinolones: *norA, norC, sdrM, arlR, arlS* Tetracyclines: *mepA, mepR, tet(38*) Efflux/others: *mgrA, LmrS, sepA*	Beta-lactams: *mecA, blaZ, blaI* Aminoglycosides: *aph(3′)-IIIa* Quinolones: *gyrA, parC, norA. norC, sdrM, arlR, arlS* Tetracyclines: *mepA, mepR, tet(38*) Fosfomycin: *murA, fosB* Trimethoprim: *dfrG* Streptothricin: *sat4* Efflux/ Others: *mgrA, LmrS, sepA*	Beta-lactams: *mecA, blaZ, blaI* Aminoglycosides: *kdpD* Quinolones: *norA, norC, sdrM, arlR, arlS* Tetracyclines: *mepA, mepR, tet(38*) Fosfomycin: *fosB* Trimethoprim: *dfrG* Efflux/others: *mgrA, LmrS, sepA*	Beta-lactams: *mecA, blaZ, blaI* Aminoglycosides: *kdpD* Quinolones: *norA, norC, sdrM, arlR, arlS* Tetracyclines: *mepA, mepR, tet(38*) Fosfomycin: *fosB* Trimethoprim: *dfrG* Efflux/others: *mgrA, LmrS, sepA*	Beta-lactams: *mecA, blaZ, blaR1, blaI* Quinolones: *norA, norC, sdrM, arlR, arlS* Tetracyclines: *mepA, mepR, tet(38*) Fosfomycin: *fosB* Efflux/others: *mgrA, LmrS, sepA*
Prophages	Three intact *S. aureus* prophages Three incomplete prophage regions	Two intact *S. aureus* prophages One incomplete prophage region	Three intact *S. aureus* prophages Three incomplete prophage regions	Three intact *S. aureus* prophages Three incomplete prophage regions	Three intact *S. aureus* prophages Two incomplete prophage regions	One intact *S. aureus* prophages One incomplete prophage region
Antiphage systems	Abi2, AbiD, Avs_II, Dodola, FS_Sma	Abi2, AbiD, AbiJ, Avs_II, Nhi, RM Type IV, RosmerTA	Abi2, AbiJ, FS_Sma, Retron_III, RM Type IIG, RosmerTA	Abi2, AbiJ, FS_Sma Retron_III, RM Type IIG, RosmerTA	Abi2, AbiJ, FS_Sma, Retron_III, RM Type IIG, RosmerTA	Abi2, FS_Sma, Retron_III, RosmerTA
Sequence Read Archive accession number	SRR30565429	SRR30565428	SRR30565427	SRR30565426	SRR30565425	SRR30565424
GenBank accession number	JBINAI000000000	JBINAH000000000	JBINAG000000000	JBINAF000000000	JBINAE000000000	JBINAD000000000

^*a*^ The complete annotation data described here is publicly available on Zenodo (https://doi.org/10.5281/zenodo.14500522).

^*ba*^ MIC, minimum inhibitory concentration based on the cutoffs set by the Clinical and Laboratory Standards Institute (5).

## Data Availability

All data from raw reads and assembled genomes are found at the accession numbers provided in Table 1, while the extended annotation data is publicly available on Zenodo (https://doi.org/10.5281/zenodo.14500522).
